# Redox-Responsive Porphyrin-Based Polysilsesquioxane Nanoparticles for Photodynamic Therapy of Cancer Cells

**DOI:** 10.3390/ijms17010056

**Published:** 2015-12-31

**Authors:** Daniel L. Vega, Patrick Lodge, Juan L. Vivero-Escoto

**Affiliations:** 1 Department of Chemistry, University of North Carolina at Charlotte, Charlotte, NC 28223, USA; dvega1@uncc.edu (D.L.V.); plodge@uncc.edu (P.L.); 2 The Center for Biomedical Engineering and Science, University of North Carolina at Charlotte, Charlotte, NC 28223, USA

**Keywords:** photodynamic therapy, photosensitizer delivery, porphyrin, stimulus-responsive materials, polysilsesquioxane nanoparticles, cancer therapy

## Abstract

The development of stimulus-responsive photosensitizer delivery systems that carry a high payload of photosensitizers is of great importance in photodynamic therapy. In this study, redox-responsive polysilsesquioxane nanoparticles (PSilQNPs) built by a reverse microemulsion approach using 5,10,15,20-tetrakis(carboxyphenyl) porphyrin (TCPP) silane derivatives as building blocks, were successfully fabricated. The structural properties of TCPP-PSilQNPs were characterized by dynamic light scattering (DLS)/ζ-potential, scanning electron microscopy (SEM) and thermogravimetric analysis (TGA). The photophysical properties were determined by UV-vis and fluorescence spectroscopy. The quantity of singlet oxygen generated in solution was measured using 1,3-diphenylisobenzofuran. The redox-responsive release of TCPP molecules was successfully demonstrated in solution in the presence of a reducing agent. The internalization of TCPP-PSilQNPs in cancer cells was investigated using laser scanning confocal microscopy. Phototoxicity experiments *in vitro* showed that the redox-responsive TCPP-PSilQNPs exhibited an improved phototherapeutic effect on cervical cancer cells compared to a non-responsive TCPP-PSilQNP control material.

## 1. Introduction

Photodynamic therapy (PDT) is an innovative minimally invasive therapy that has great potential to selectively destroy malignant cells while sparing normal cells [1,2,3,4,5]. PDT is currently approved for the treatment of various types of cancers, including lung, head and neck, esophageal and cervical cancers. PDT uses photosensitizer (PS) agents that will localize, ideally, in a specific tumor tissue, at which point irradiation with light of the appropriate wavelength will activate the PS. Upon activation with light, the PS molecule interacts with molecular oxygen to generate singlet oxygen (^1^O_2_) and reactive oxygen species (ROS), leading to the destruction of cancer cells through apoptosis or through necrosis [2,3,6,7]. Despite the favorable advantages of PDT, the clinical application of this therapeutic approach has been limited. Several reasons can account for that such as the poor penetration of light in tissue and its dependence on the presence of oxygen [2,8]. In addition, there are several limitations associated specifically to the PS agents such as the development of non-specific skin phototoxicity, poor water solubility and inefficient delivery to tumor tissues [9,10,11]. Therefore, novel delivery systems are necessary to improve the specificity and enhance the phototherapeutic efficacy of PDT.

**Scheme 1:**
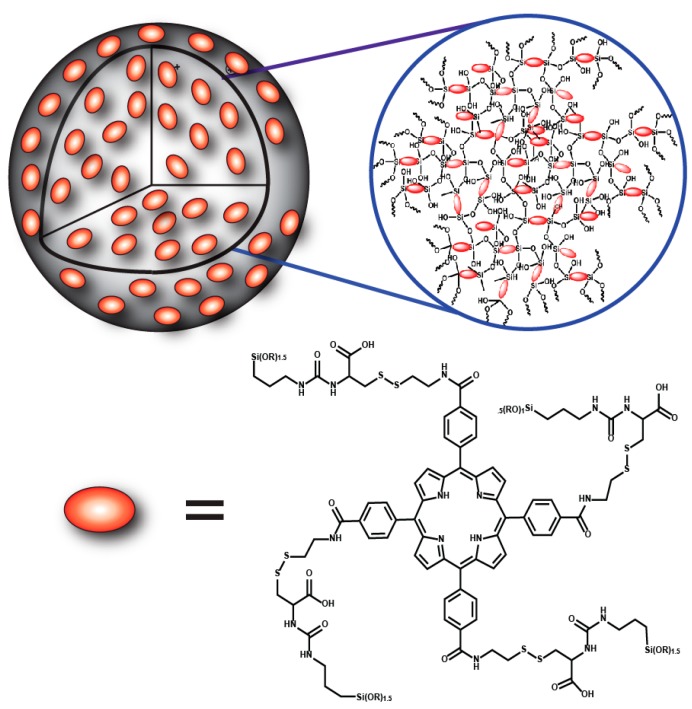
Schematic representation of the redox-responsive porphyrin-based polysilsesquioxane nanoparticle (PSilQNP) platform developed in this work. The framework of the nanoparticle is made of tetrakis(carboxyphenyl) porphyrin (TCPP)-based monomers, which contains a disulfide bridge and silica bonds as connecting units.

**Scheme 2:**
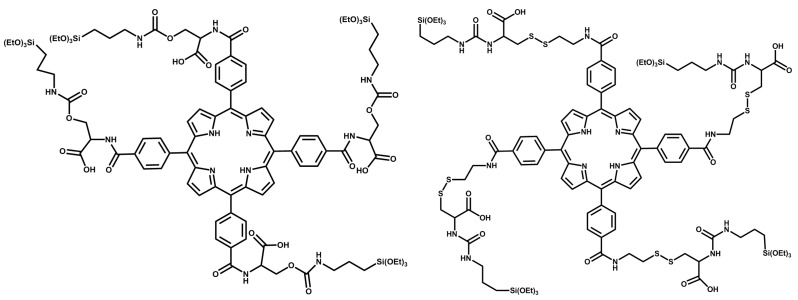
Two TCPP-based monomers are synthesized in this work, control TCPP (C-TCPP) (**left**) and redox-responsive TCPP (RR-TCPP) (**right**). Both molecules contain triethoxysilane groups that can be polymerized to afford PSilQNPs and carboxylic acid moieties that can be used for further functionalization. Moreover, RR-TCPP has disulfide bonds that are cleaved under high reducing conditions, such as those found in cancer cells.

Nanoparticle-based PS delivery platforms have emerged as alternative approaches to overcome some of the delivery issues of PSs. Nanoparticles offer several advantages as PS delivery systems: they can carry large payloads of PS molecules; their surfaces and compositions can be tailored to develop multifunctional systems; and, due to their sizes in the nanoscale regime, these materials are known to accumulate at tumor sites by the so-called enhanced permeability and retention (EPR) effect [12,13,14,15,16,17,18]. Several groups have already demonstrated that PS-loaded nanocarriers could enhance the tumor target specificity and therapeutic efficacy in cancer treatment [19,20,21,22]. In addition, nanoparticulate approaches have been used for combination therapy including PDT [23,24,25,26]. Hayashi and coworkers recently reported on the synthesis of an iodinated silica/porphyrin hybrid nanoparticle. This platform was successfully applied for the PDT/Photothermal therapy (PTT) combination treatment of multiple myeloma *in vivo* [25]. Despite the encouraging results using nanoparticle-based PS delivery systems, there are two main problems that prevent nanoparticles from reaching their highest potentials as PS carrier platforms. One issue is the potential trapping of the produced oxidative species (^1^O_2_ and ROS) inside the nanoparticle due to the presence of the nanocarrier’s matrix, which slows down or completely prevents the out-diffusion of the generated oxidative species [16]. Moreover, another hurdle is the self-quenching of PSs encapsulated inside the nanoparticles, which occurs because of their spatial proximity [27]. This effect is enhanced in PS delivery platforms that contain large number of PSs [28,29,30]. Both limitations would largely reduce the phototoxic effect of PSs against cancer cells. One of the strategies that has been explored to overcome these issues is the development of stimuli-responsive nanoparticle-based platforms that can degrade upon specific conditions such as low pH, highly reducing environments, *etc*. These materials increase the phototherapeutic efficacy in tumor tissues after the material has dissociated inside cancer cells [29,30,31,32,33]. Our group and others have explored the use of disulfide bonds to develop redox-responsive PS delivery systems. The introduction of disulfide bonds enables the PS delivery nanocarrier to release its payloads efficiently in intracellular reductive environments [34,35,36,37,38]. Huh and coworkers reported on the synthesis and application *in vitro* and *in vivo* of the PDT agent pheophorbide A (PheoA) conjugated with glycol chitosan (GC) polymer via reducible disulfide linkages [34]. The developed polymer self-assembled forming core-shell spherical nanoparticles (CNPs) (PheoA-ss-CNPs) about 200 nm in diameter. The photoactivity and therapeutic efficacy of this platform was compared with non-reducible NPs (PheoA-CNPs) *in vitro*. The reducible NPs showed rapid cellular uptake and significantly higher phototoxicity than the non-reducible NPs due to the dissociation of NPs in the intracellular reductive environment. The *in vivo* imaging results showed that the reducible NPs selectively accumulated to the tumor site through the EPR effect. The results of *in vivo* therapeutic efficacy studies in tumor-bearing mice showed that a significantly decreased tumor volume was observed for PDT with PheoA-ss-CNPs. Durand and coworkers reported on the development of biodegradable two-photon PDT medical devices using disulfide linkers. In this work, bridged silsesquioxane (BS) NPs were used as platforms to incorporate disulfide bridges, two-photon electron donor (diamino diphenylbutadiene, 2PS) agents or zinc-5,10,15,20-tetra(propargyloxyphenyl) porphyrins (POR) [35]. The BSNPs had a high loading of 2PS (28 *w*t %) and POR (10–14 *w*t %). Moreover, these NPs were degraded in the presence of a reducing agent (2 mM mercaptoethanol). The photo imaging and therapeutic properties of this platform was successfully evaluated *in vitro* using breast cancer MCF-7 cells. Our group has also reported on the synthesis, characterization and *in vitro* application of redox-responsive nanoparticles containing the protoporphyrin-IX (PpIX) molecule as a PS agent (RR-PpIX-PSilQNPs) [37]. This platform showed the redox-responsive release capabilities of PSs in the presence of a reducing agent. Moreover, phototoxic evaluation of RR-PpIX-PSilQNPs in HeLa cells showed higher phototoxicity than that of a control sample (C-PpIX-PSilQNPs) that did not contain disulfide bonds in the network. We hypothesized that the enhancement in the phototherapeutic effect for RR-PpIX-PSilQNPs was due to selective release of PpIX molecules after internalization in cancer cells. This hypothesis was later corroborated by confocal microscopy using a double-labeled core-shell nanoparticulate approach [38]. In this study, we report on the synthesis, characterization and *in vitro* application of a redox-responsive PSilQ platform containing tetrakis(carboxy)phenyl porphyrin (TCPP) (Scheme 1). Two building block molecules based on TCPP, one control (C-TCPP) and one redox-responsive (RR-TCPP) derivatives (Scheme 2), were synthesized in multi-step reactions. The RR-TCPP ligand incorporates a disulfide bond that is cleaved under reducing conditions such as those found inside of cancer cells. Both TCPP derivatives include triethoxysilane groups, which, after condensation in a reverse microemulsion reaction, afforded the PSilQNPs. The structural properties of these TCPP-based PSilQNPs showed that PSilQNPs were synthesized with sizes of 50–70 nm in diameter and high contents of TCPP, on the order of 120–150 µmol per g of PSilQNPs. Moreover, we have shown that once the RR-TCPP-PSilQNPs have been internalized in the cells, the redox-responsive PSilQ platform increases phototoxicity in comparison to the C-TCPP-PSilQNPs material.

## 2. Results and Discussion

### 2.1. Synthesis and Characterization of Redox-Responsive Tetrakis(Carboxyphenyl) Porphyrin (RR-TCPP) and Control Tetrakis(Carboxyphenyl) Porphyrin(C-TCPP) Silane Derivatives

**Scheme 3:**
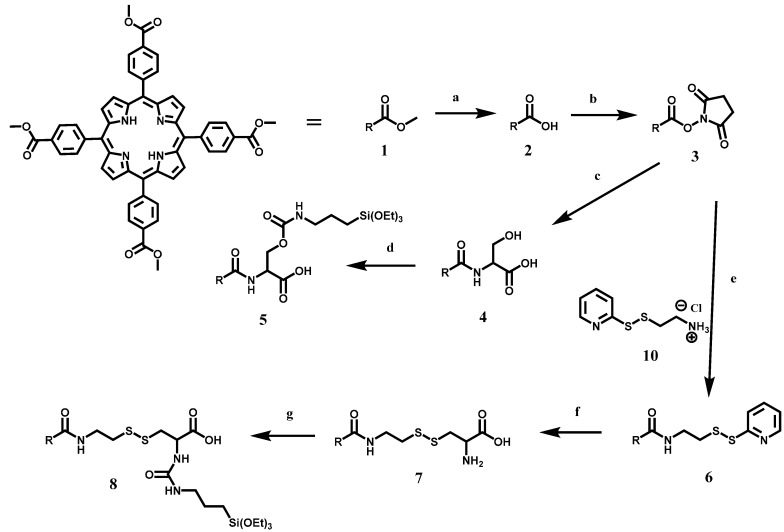
Schematic representation of the synthesis of C-TCPP and RR-TCPP silane derivatives. For simplification, the tetrakis(phenyl) porphyrin is represented as the R group. See details of the experimental conditions for each of the reactions in the Experimental Section. The synthesis of 5,10,15,20-tetrakis(4-carbomethoxyphenyl) porphyrin (TCM_4_PP) (**1**) is carried out by the condensation of pyrrole and benzaldehyde; (**a**) Hydrolysis of **1** under basic conditions afforded TCPP (**2**); (**b**) Compound **2** can be activated toward acyl nucleophilic substitution by the formation of the ester bond with *N*-hydroxysuccinimide (NHS) (**3**); (**c**) For the synthesis of C-TCPP, compound **3** underwent an acyl nucleophilic substitution with serine to produce TCPP-Serine (**4**); (**d**) Finally, **4** reacted with triethoxysilane propyl isocyanate (TES-PI) to obtain C-TCPP (**5**); (**e**) To produce RR-TCPP, compound **3** also underwent acyl nucleophilic substitution with pyridyl disulfide cysteamine (PDSCA) (**10**) to form TCPP-PDSCA (**6**); (**f**) Compound **7** is synthesized by the disulfide exchange reaction between **6** and cysteine; (**g**) Lastly, RR-TCPP (**8**) is produced by reacting **7** with TES-PI.

To fabricate the TCPP-PSilQNPs developed in this work, two novel TCPP silane derivatives were synthesized and characterized (Scheme 2 and Scheme 3). First, the synthesis of 5,10,15,20-tetrakis(4-carbomethoxyphenyl) porphyrin (TCM_4_PP; **1**) was carried out through the reaction of benzaldehyde and pyrrole in propionic acid at 150 °C. TCM_4_PP then underwent hydrolysis under basic conditions in tetrahydrofuran (THF)/Ethanol (EtOH) to afford TCPP (**2**). A distinct change in the stretching vibration of the carbonyl group from the methyl ester (1720 cm^−1^) to the carbonyl corresponding to the carboxylic acid (1694 cm^−1^), along with the disappearance of the methyl group in ^1^H- and ^13^C-NMR demonstrated the successful synthesis of TCPP. The next synthetic step was the conjugation of TCPP with *N*-hydroxysuccinimide (NHS) in the presence of 1-ethyl-3-(3-dimethylaminopropyl) carbodiimide (EDC) to afford TCPP-succinimide ester (SE) (**3**). The TCPP-SE derivative includes a succinimide ester, which is an excellent leaving group for the nucleophilic acyl substitution with amines to afford the corresponding amides. The synthesized TCPP-SE molecule showed a diagnostic stretching vibration in IR corresponding to the ester and succinimide groups (1736, 1770 and 1803 cm^−1^). In addition, the appearance of the ethylene groups of the succinimide in ^1^H- and ^13^C-NMR provided further evidence for the successful synthesis of TCPP-SE. To afford the C-TCPP silane derivative, **3** was reacted with serine in dimethylsulfoxide (DMSO) followed by aqueous work-up in acidic conditions to afford the amino acid form of TCPP, TCPP-Serine (**4**). The amine group of serine is a stronger nucleophile than the alcohol group, which allowed the exclusive synthesis of the amide bond, but not of the ester derivative. The disappearance of the succinimide peaks from NHS and the appearance of serine peaks in IR and ^1^H-NMR confirmed the synthesis of TCPP-Serine. It is important to point out that compounds **1**, **2**, **3** and **4** were also confirmed with matrix-assisted laser desorption/ionization-time of flight (MALDI-TOF) mass spectrometry (see Experimental Section). Lastly, the C-TCPP silane derivative was synthesized by reacting TCPP-Serine with triethoxysilane propyl isocyanate (TES-PI) under N_2_ atmosphere in anhydrous dimethylformamide (DMF) for 22 h. This was followed by aqueous work-up in acidic conditions to afford C-TCPP (**5**). The stretching vibrations in the IR spectrum for the carbonyl group (1706 cm^−1^), along with the appearance of the Si–C (1233 cm^−1^) and Si–O (1016 cm^−1^), are indications of the successful synthesis of C-TCPP.

The synthesis of the RR-TCPP silane derivative was carried out following the steps depicted in Scheme 3. First, TCPP-SE reacted with pyridyl disulfide cysteamine (PDSCA; **10**) and Et_3_N in DMF at 80 °C to afford TCPP-PDSCA (**6**). ^1^H-NMR confirmed the synthesis of TCPP-PDSCA, the peaks in the ^1^H-NMR for the succinimide group are no longer present; moreover, the aromatic protons corresponding to the pyridine group are observed. TCPP-PDSCA is further reacted through a disulfide exchange reaction with cysteine in DMF at 60 °C to obtain TCPP-Cysteine (**7**). The disappearance of the peaks in ^1^H-NMR corresponding to pyridine and the appearance of the protons associated with cysteine suggested that the disulfide reaction was successful. Finally, TCPP-Cysteine reacted with TES-PI in anhydrous DMF under N_2_ atmosphere to afford RR-TCPP (**8**). The stretching vibrations in the IR spectrum provide evidence for the synthesis of RR-TCPP. The IR shift for the carbonyl group (1714 cm^−1^) along with the appearance of the Si–C (1222 cm^−1^) and Si–O (1019 cm^−1^) are indicative of the successful synthesis of RR-TCPP.

### 2.2. Singlet Oxygen Generation of TCPP-Serine (**4**) and TCPP-EtSH (**9**)

**Figure 1:**
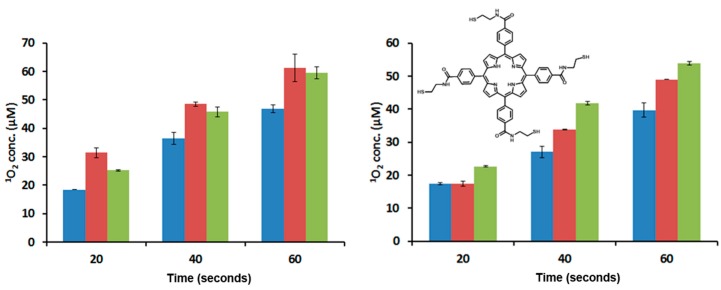
Determination of ^1^O_2_ production by compounds **2** (**blue**), **4** (**red**) and **9** (**green**) after irradiation with white (**left**) and red (**right**) light. Insert: Chemical structure of **9**. Error bars represent the standard deviation of three independent experiments.

PDT is dependent on the presence of molecular oxygen. This suggests that ^1^O_2_ generated by the photosensitization of molecular triplet oxygen is the principal toxic species formed during PDT. Therefore, the generation of singlet oxygen is extremely crucial to the success of PDT, and one of the first tests performed on new PSs is to probe their abilities for ^1^O_2_ generation [39]. The photophysical properties of porphyrins, such as quantum yields, lifetimes and ^1^O_2_ generation, are mainly affected by core modifications with the incorporation of transition metals and/or the replacement of one or more of the porphyrin pyrrolic nitrogens with other heteroatoms [40,41]. However, modifications on the *meso* phenyl rings with heavy atoms in molecules like tetraphenylporphyrin have also shown enhancement in the generation of ^1^O_2_ [42]. To evaluate whether the chemical modifications of TCPP (**2**) with serine and cysteamine cause an effect on the ^1^O_2_ generation, we measured the amount of ^1^O_2_ produced by TCPP-Serine (**4**) and TCPP-EtSH (**9**). TCPP-EtSH is the PS agent produced after RR-TCPP is reduced in the presence of a reducing agent (see insert in Figure 1); the Experimental Section shows the details for the synthesis of **9**. The ^1^O_2_ production is measured by using a singlet oxygen chemical probe (1,3-diphenylisobenzofuran, DPBF). DPBF is a singlet oxygen scavenger that reacts in a Diels–Alder [[4 + 2]-cycloaddition with the singlet oxygen generated by the excited PS. DPBF usually absorbs light at 419 nm; however, after the reaction with ^1^O_2_ the resulting product does not absorb light at that wavelength [43]. Samples of **2**, **4** and **9** were prepared in DMF (2.5 μM) together with DPBF (5 μM). The samples were illuminated using white (400–700 nm; 41 mW/cm^2^) or red (630–700 nm; 89 mW/cm^2^) light at different times. The data show that there is an increased in the generation of ^1^O_2_ by **4** and **9** as compared with **2** after irradiation with white light. Nevertheless, there were no statistically significant differences between **4** and **9** (Figure 1). When the TCPP derivatives were irradiated with red light, a slightly difference in the generation of ^1^O_2_ was observed following the trend of **9** > **4** > **2** (Figure 1). The most important conclusion from the ^1^O_2_ generation data for the goal of this work is that there was not a dramatic reduction in the ^1^O_2_ production after the functionalization of TCPP molecule. Additional experiments, which are out of the scope of this work, need to be done to find out whether the differences in ^1^O_2_ generation from compounds **4** and **9** are due to solubility and/or electronic effects associated with the chemical modifications of **2**.

### 2.3. Synthesis and Structural Characterization of RR-TCPP-PSilQ and C-TCPP-PSilQ Nanoparticles

**Figure 2:**
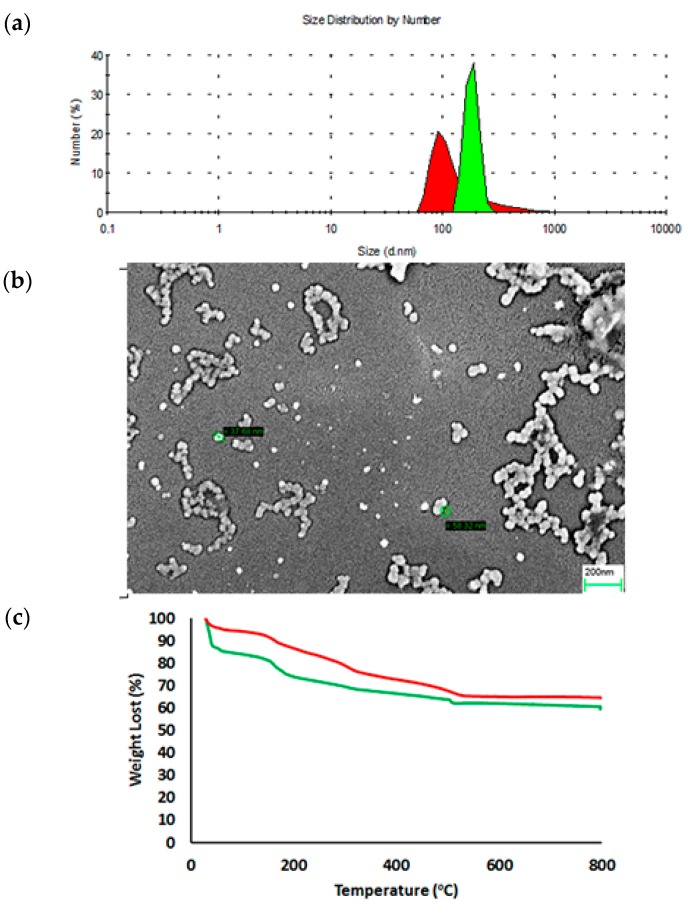
Structural properties of C-TCPP- and RR-TCPP-PSilQNPs. (**a**) Dynamic light scattering plot of C-TCPP- (green) and RR-TCPP-PSilQNPs (red); (**b**) SEM image of C-TCPP-PSilQNPs (green circles show the diameter of individual nanoparticles); Scale bar = 200 nm; and (**c**) Thermogravimetric analysis (TGA) plot of C-TCPP- (green) and RR-TCPP-PSilQNPs (red).

The PSilQNPs in this work were synthesized by following a reverse microemulsion method composed of a quaternary system. Reverse phase microemulsions consist of water droplets in the nanoscale regimen, which are stabilized by a surfactant and/or co-surfactant in an organic phase [44]. The quaternary system consists of triton X-100, 1-hexanol, cyclohexane and C-TCPP or RR-TCPP, which are used as surfactant, co-surfactant, organic solvent and silica precursor, respectively. To synthesize the TCPP-based PSilQNPs, the silica precursor is dissolved in water in the presence of a base (NH_4_OH) to accelerate the polymerization reaction. Previous experience in our group with porphyrin-based silica precursors has shown several challenges to dissolve these silica precursors in aqueous solutions [37]. However, in the case of C-TCPP and RR-TCPP molecules, the presence of carboxylic acid groups facilitates this step because they can be deprotonated under basic conditions affording carboxylates, which are more soluble in aqueous solutions. The solution containing the TCPP silane derivative is later added to the organic phase, which is composed of triton X-100, 1-hexanol and cyclohexane. The reverse microemulsion reaction is carried out for 24 h at room temperature (RT). After that, the TCPP-based PSilQNPs are obtained by centrifugation after the material has been crashed down with EtOH. The structural properties of these PSilQNPs were characterized by DLS, ζ-potential, SEM and TGA (Figure 2 and Table 1). The DLS showed that the hydrodynamic diameter of C-TCPP- and RR-TCPP-PSilQNPs is 183.8 ± 10.5 and 144.3 ± 15.0 nm, respectively. The hydrodynamic diameter is around two times bigger than what it is observed by SEM. The SEM micrographs showed diameters for C-TCPP- and RR-TCPP-PSilQNPs of 60.1 ± 9.2 and 57.5 ± 7.7 nm (see Figure 2 and Figure S1), respectively. The difference between the hydrodynamic diameter and the particle size found by SEM may be due to the influence of the solvent, the aggregation of the PSilQNPs and/or their ability to swell after adsorption of water molecules, hydrogel-like behavior [45]. However, it is important to point out that the colloidal stability of this TCPP-PSilQNPs has increased dramatically with what we have reported before for porphyrin-based PSilQNPs [37]. The improvement in the colloidal stability is most likely due to the presence of the carboxylate groups on the surface of the nanoparticles. The ζ-potential for C-TCPP and RR-TCPP-PSilQNPs in PBS (1 mM, pH 7.4) was −39.7 ± 2.8 and −44.5 ± 2.5 mV, respectively. The ζ-potential measurements confirmed that the surface of the PSilQNPs is negatively charged, as mentioned above, due to the presence of carboxylates groups on the surface of the nanoparticles. The amount of aromatic organic content by TGA for C-TCPP and RR-TCPP PSilQNPs was determined to be 10.1 and 11.3%*w*t., respectively. These values were determined by using the weight lost between 350 and 800 °C, which is the region where TCPP losses more than 95%*w*t. of its organic content (Figure S2). Based on this data the amount of TCPP loaded to C-TCPP and RR-TCPP-PSilQNPs was calculated as 127.7 and 142.0 µmol per g of PSilQNPs, respectively. Nevertheless, the values obtained through UV-visible spectroscopy for the loading of TCPP were 80.3 and 89.3 µmol per g of PSilQNPs for C-TCPP and RR-TCPP-PSilQNPs, respectively. The difference can be accounted by the aggregation of TCPP molecules inside PSilQNPs, which prevents the absorption of light as compared with individual units in solution.

**Table 1:** Structural properties of control tetrakis(Carboxyphenyl) porphyrin (C-TCPP)- and redox-responsive tetrakis(carboxyphenyl) porphyrin (RR-TCPP)-polysilsesquioxane nanoparticles (PSilQNPs).

Sample	Diameter (nm) * *n* = 3	PDI	ζ-Potential (mV) * *n* = 3	Aromatic Content (%)	Loading of TCPP (µmol/g)
C-TCPP-PSilQNPs	183.8 ± 10.5	0.39	−39.7 ± 2.8	10.1	127.7
RR-TCPP-PSilQNPs	144.3 ± 15.0	0.33	−44.5 ± 2.5	11.3	142.0

* Data measured in phosphate buffer solution (1 mM; pH 7.4)/Concentration of PSilQNPs = 0.1 mg/mL; PDI = Polydispersity index.

### 2.4. Photophysical and Photochemical Properties of C-TCPP- and RR-TCPP-PSilQ Nanoparticles

**Figure 3:**
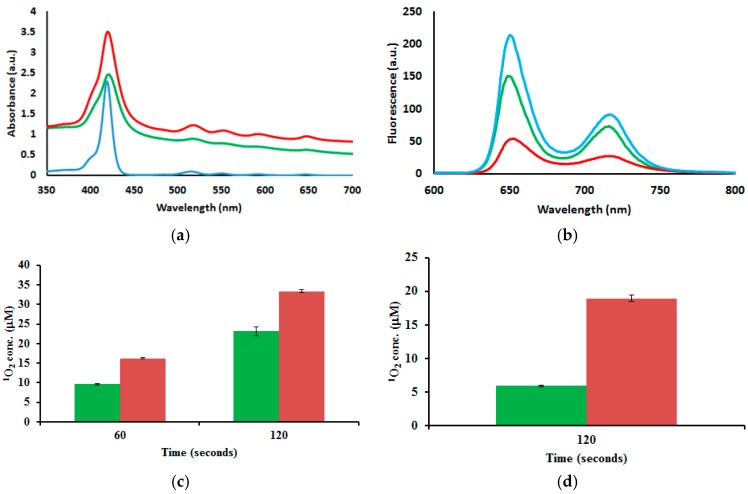
Photophysical and photochemical properties of C-TCPP- and RR-TCPP-PSilQNPs. (**a**) UV-vis spectroscopy of **2** (blue), C-TCPP- (green) and RR-TCPP-PSilQNPs (red); and fluorescence spectroscopy (**b**) of **2** (blue), C-TCPP- (green) and RR-TCPP-PSilQNPs (red). Determination of ^1^O_2_ production by C-TCPP- (green) and RR-TCPP-PSilQNPs (red) after irradiation with white (**c**) and red (**d**) light. Error bars represent the standard deviation of three independent experiments.

UV-vis spectroscopy showed the successful encapsulation of TCPP in the C-TCPP and RR-TCPP PSilQNP framework as shown by the Soret band at 420 nm and the Q bands at 518, 552, 592 and 648 nm (Figure 3). These bands are similar to the parent TCPP molecule; which are Soret band at 419 nm and the Q bands at 515, 551, 590 and 646 nm. Fluorescence spectroscopy measurements show that the emission spectra of both the C-TCPP- and RR-TCPP-PSilQ materials is also similar to TCPP without any significant spectral shifts (Figure 3). These results suggest that TCPP was successfully incorporated into PSilQNPs without major influence in the photophysical properties of the parent porphyrin. In addition, the production of ^1^O_2_ by C-TCPP- and RR-TCPP-PSilQNPs was determined using the singlet oxygen probe DPBF as described in Section 2.2. Interestingly, the amount of ^1^O_2_ generated by the nanoparticles has been dramatically reduced, even though they have the same concentration of TCPP molecules as the experiment depicted in Section 2.2 (Figure 3). To obtain meaningful values from the ^1^O_2_ test, we had to increase the irradiation time for both white and red light. This clearly indicates that the TCPP molecules incorporated in the framework of the PSilQNPs do not generate singlet oxygen efficiently [34,37].

### 2.5. Stimuli-Responsive Properties of RR-TCPP-PSilQ Nanoparticles

**Figure 4:**
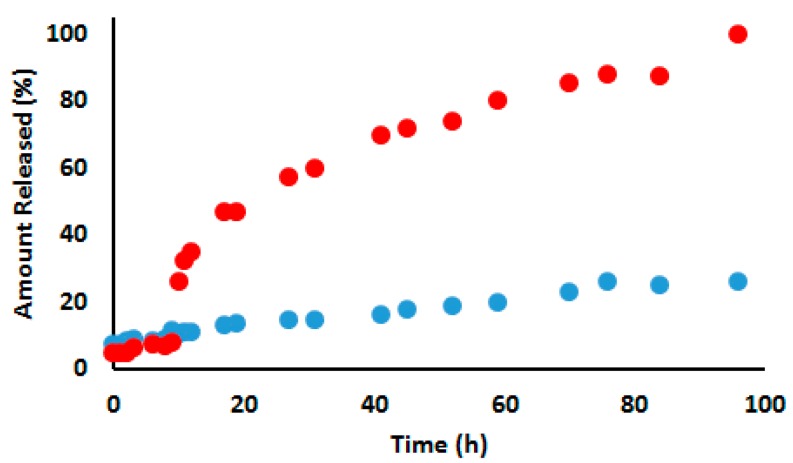
Release profile of TCPP-EtSH from RR-TCPP-PSilQNPs incubated in the presence (**red circles**) and the absence (**blue circles**) of the reducing agent dithiothreitol. Dithiothreitol (DTT) was added at time equal 9 h.

The RR-TCPP-PSilQNPs were designed to be stable under normal physiological conditions, but they can be readily dissociated to release the TCPP-EtSH (**9**) molecules upon the reductive cleavage of the disulfide bonds by reducing agents such as dithiothreitol (DTT), such as glutathione and cysteine [34,37]. To evaluate the degradation ability of RR-TCPP-PSilQNPs under high reducing conditions, we measured the release of **9** in solution in the presence and the absence of a reducing agent. The release experiment revealed that RR-TCPP-PSilQNPs are stable in the absence of reducing agents (first 9 h), with only 10% or less background released (Figure 4). However, after the addition of a DTT solution (10 mM), TCPP-EtSH molecules were immediately released from the RR-TCPP-PSilQNPs reaching 25% release in the first hour and a half-life (*t*_1/2_) of approximately 23 h. In this experiment, more than 80% of the TCPP-EtSH molecules were released after 59 h of incubation with DTT. By contrast, in our control experiment, the RR-TCPP-PSilQNPs that are not incubated with DTT solution showed less than 18% release after 96 h of incubation. The total amount released after 96 h in the presence of DTT was 40.9 µmol TCPP-EtSH per g of RR-TCPP-PSilQNPs. The material was completely degraded after eight days of incubation in the presence of DTT agent (data not shown).

### 2.6. In Vitro Phototoxicity of C-TCPP- and RR-TCPP-PSilQ Nanoparticles

**Figure 5:**
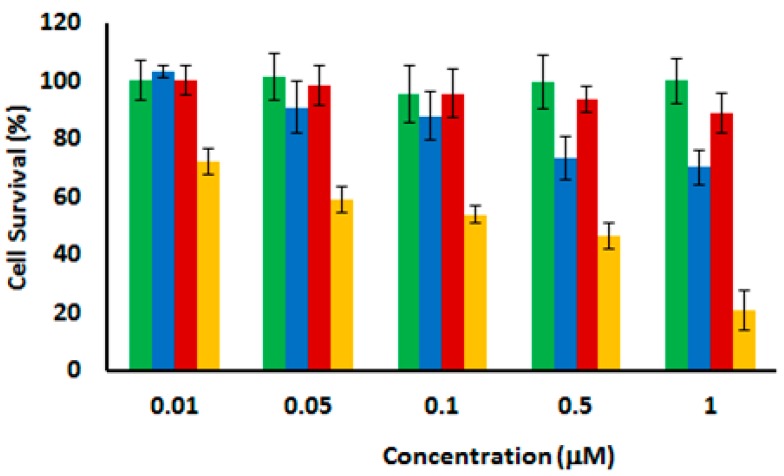
Phototoxicity of C-TCPP-PSilQNPs (**green**) and RR-TCPP-PSilQNPs (**red**) in the absence of light, and C-TCPP-PSilQNPs (**blue**) and RR-TCPP-PSilQNPs (**orange**) after light exposure (630–700 nm; 89 mW/cm^2^; 20 min). Error bars represent the standard deviation of three independent experiments with six repetitions each.

The phototocytotoxicity of C-TCPP- and RR-TCPP-PSilQNPs in human cervical cancer (HeLa) cells was investigated by the MTS assay. HeLa cells were inoculated at different concentrations for 24 h with each material and then irradiated with red light (630–700 nm; 89 mW/cm^2^) for 20 min. The “dark” cytotoxicity, samples not irradiated with light, was also determined at the same concentrations of PSilQNPs as the control experiment. Figure 5 shows the cell survival of HeLa cells that have been incubated for 24 h after light irradiation. The cytotoxicity of the samples in absence of light showed that both PSilQNPs are non-cytotoxic at the concentrations evaluated in this experiment. Nevertheless, the cell viability decreased in the presence of both C-TCPP- and RR-TCPP-PSilQNPs after light exposure. Of note, the decrease in cell survival is more noticeable with RR-TCPP-PSilQNPs as an indication of the capability of this material to transport and deliver PS agents in a more efficient way. Based on previous works from the literature, we hypothesized that TCPP-EtSH molecules are released in monomeric form under intracellular reducing conditions and without any loss of photoactivity [34,37,38]. The half maximal inhibitory concentration (IC_50_) for RR-TCPP-PSilQNPs after irradiation with red light is around 0.1 µM. The internalization of RR-TCPP-PSilQNPs in HeLa cells was confirmed by laser scanning confocal microscopy (Figure S3). Overall, the *in vitro* data show that the RR-TCPP-PSilQNPs can efficiently transport and deliver the TCPP-EtSH molecules, thereby avoiding ^1^O_2_ trapping in the nanoparticle framework and self-quenching. As a result, the phototoxic effect on HeLa cells has been improved.

## 3. Experimental Section

### 3.1. Materials and Methods

All of the reagents were purchased from Aldrich and used without further purification. Thermogravimetric analysis (TGA) was determined using a Mettler Toledo TGA/SDTA851 instrument (Mettler-Toledo AG Analytical, Schwersenbach, Switzerland) with a platinum pan and a heating rate of 1.0 °C/min from 25.0 to 800.0 °C under a nitrogen atmosphere. The sample was held at 800.0 °C for 3 h to make sure that all the organic material had been calcined. A Raith 150 Field Emission Scanning Electron Microscope (SEM) (Raith America Inc., New York, NY, USA) was utilized to measure the particle size and shape of the materials. Nanoparticle samples were suspended in methanol in preparation for the SEM. Dynamic light scattering (DLS) and ζ-potential measurements were carried out using a Malvern Instrument Zetasizer Nano (red laser 633 nm) (Malvern Instrument Ltd., Malvern, UK). The amount of TCPP molecules loaded into the PSilQNPs was quantified by UV-vis spectroscopy (Varian, Cary 300 Bio UV/vis spectrometer) (Varian, Sidney, Australia). The photophysical properties of TCPP-based PSilQNPs were determined using UV-vis and fluorescence (Varian, Cary Eclipse fluorescence spectrometer) (Varian, Sidney, Australia).

### 3.2. Synthesis of 5,10,15,20-Tetrakis(carbomethoxy)phenyl Porphyrin (TCM_4_PP) (**1**)

To synthesize **1**, 2.294 g of methyl-4-formyl benzoate (14.0 mmol) was added to propionic acid (150.0 mL), this solution was stirred and heated at 151 °C. Then, 970 µL (14.0 mmol) of pyrrole was added and the solution was allowed to reflux for 1 h. The product was purified by washing with cold methanol and filtered to obtain deep purple crystals. The product was dried under high vacuum and stored at room temperature. Yield: 651 mg, 22.0%. IR: 1720 cm^−1^; ^1^H-NMR: (300 MHz; CDCl_3_): δ_H_, ppm (s, 12H, 4.1), (d, 8H, 8.3), (d, 8H, 8.4), (s, 8H, 8.8); ^13^C-NMR: (300 MHz; CDCl_3_): δ_C_, ppm 52.60, 119.51, 128.11, 129.87, 131.21, 134.64, 146.72, 167.35 (ester); Calculated mass for **1**: 846.90 g/mol; MS (MALDI positive ion): *m*/*z* 847.34 [M + 1]^+^, 848.67 [M + 2]^+^, 849.38 [M + 3]^+^.

### 3.3. Synthesis of Tetrakis(carboxy)phenyl Porphyrin (TCPP) (**2**)

To synthesize **2**, 500 mg of **1** (590 µmol) was added to a mixture of THF:EtOH (30 mL; 1:1 *v*/*v*) containing 4 mL of KOH (2 M). The mixture was stirred at 70 °C for 24 h. The product was obtained by rota-evaporating the solvent mixture and dissolving in 300 mL of water followed by the addition of 850 µL HCl (37%/*v*) to afford precipitation of **2**. The dark blue crystals were filtered, dried under high vacuum and stored at room temperature. Yield: 452 mg, 96.8%. IR: 1694 cm^−1^; ^1^H-NMR: (300 MHz, *d*_6_-DMSO): δ_H_, ppm (q, 16H, 8.26–8.39), (s, 8H, 8.85); ^13^C-NMR: (500 MHz, *d*_6_-DMSO): δ_C_, ppm 119.84, 128.43, 129.95, 131.00, 132.16, 134.99, 145.95 167.97 (acid); Calculated mass for **2**: 790.79 g/mol; MS (MALDI): *m*/*z* [M], 790.9; Absorbance (DMF): Soret band (λ_max_ = 419 nm, ε = 399,000 M^−1^·cm^−1^, *r*^2^ = 0.9989).

### 3.4. Synthesis of Succinimide Ester of TCPP (TCPP-SE) (**3**)

To synthesize **3**, 455 mg of **2** (575 µmol) was combined with 993 mg NHS (8.6 mmol), 422 mg dimethylaminopyridine (DMAP) (3.5 mmol) and 1.1 g EDC (5.7 mmol) in a mixture of dichloromethane (DCM):DMSO (110 mL; 1.75:1 *v*/*v*). This solution was stirred for 2 h in an ice bath. After that, the mixture was removed from the ice bath and stirred for another 48 h at room temperature. The succinimide ester TCPP-based molecule was obtained by precipitation in aqueous solution containing 20% EtOH. The solid was washed several times with the same ethanolic solution and dried using a lyophilizer. The final product was stored at −20 °C. Yield: 651 mg, 96%. IR: 1736 cm^−1^ (ester), 1770 cm^−1^ (NHS), 1803 cm^−1^ (NHS); ^1^H-NMR: (300 MHz, *d*_6_-DMSO): δ_H_, ppm (s, broad, 16H, 3.00), (q, 16H, 8.51–8.58), (s, 8H, 8.95); ^13^C-NMR: (500 MHz, *d*_6_-DMSO): δ_C_, ppm 26.15 (methylene), 162.64 (ester), 171.01 (NHS). Calculated mass for **3**: 1175.13 g/mol; MS (MALDI positive ion): *m*/*z* 1176.04 [M + 1]^+^, 1177.42 [M + 2]^+^, 1178.56 [M + 3]^+^.

### 3.5. Synthesis of TCPP Serine Derivative (TCPP-Serine) (**4**)

To synthesize **4**, 300 mg of **3** (255 µmol) was combined with 203 mg of l-serine hydrochloride (1.9 mmol) and 437 µL *N*,*N*-Diisopropylethylamine (DIPEA) (2.5 mmol) in DMSO (25 mL). The serine was first dissolved in water (3.75 mL) before adding to DMSO. The mixture was stirred for 48 h at 100 °C. After that, the serine derivative was purified by precipitation in aqueous solution containing 25% EtOH followed by the addition of 180 µL HCl (37%/*v*). The blue crystals were washed several times with the same solution and dried using a lyophilizer. The final product was stored at −20 °C. Yield: 243 mg, 84.0%. IR: 1634 cm^−1^ (amide); ^1^H-NMR: (300 MHz; *d*_6_-DMSO): δ_H_, ppm (d, 8H, 3.92–3.94), (m, 4H, 4.63–4.69); ^13^C-NMR: (300 MHz; *d*_6_-DMSO): δ = 56.41, 61.86 (aliphatic carbons), 166.93 (amide), 172. 57 (acid); Calculate mass for **4**: C_60_H_50_N_8_O_16_, 1139.10 g/mol; MS (MALDI): *m*/*z* [M] 1139.10; Absorbance (DMF): Soret band (λ_max_ = 419 nm, ε = 324,600 M^−1^·cm^−1^, *r*^2^ = 0.9994).

### 3.6. Synthesis of Control TCPP Silane Derivative (C-TCPP) (**5**)

To synthesize C-TCPP (**5**), 170 mg of **4** (149 µmol) was combined with 155 µL of 3-(triethoxysilyl)propyl isocyanate (TES-PI) (626 µmol) and 183 µL triethylamine (Et_3_N) in anhydrous *N*,*N*-Dimethylformamide (DMF) (10 mL) and stirred for 2 h in an ice bath under N_2_ atmosphere. The mixture was removed from the ice bath and stirred at room temperature for another 20 h under N_2_ atmosphere. The control ligand was obtained by precipitation in 80 mL of water followed by the addition of 150 µL of HCl (37%/*v*). The black powder was washed several times with the same ethanolic solution and dried using a lyophilizer. The final product was stored at −20 °C. Yield 219 mg, 69.0%. IR: 1016 cm^−1^ (Si–O), 1233 cm^−1^ (Si–C), 1706 cm^−1^ (carbamate).

### 3.7. Synthesis of TCPP-Pyridine Disulfide Cysteamine (TCPP-PDSCA) (**6**)

To synthesize **6**, compound **3** (314 mg, 267 mmol) was combined with **10** (386 mg, 1.7 mol) and Et_3_N (292 µL, 2.1 mmol) in DMSO (6.5 mL) and stirred at 80 °C for 3 days. The product was purified by precipitation in aqueous solution containing 20% EtOH. The brown powder was washed several times with the same ethanolic solution and dried using a lyophilizer. The final product was stored at −20 °C. Yield: 195 mg, 50.0%. IR: 1605 cm^−1^ (aromatic), 1638 cm^−1^ (amide); ^1^H-NMR: (300 MHz; *d*_6_-DMSO): δ_H_, ppm (t, 8H, 2.91–3.21), (m, 8H, 3.62–3.89), (t, 4H, 7.24–7.32), (d, 4H, 7.58–7.65), (t, 4H, 7.76–7.85), (q, 16H, 8.16–8.39), (d, 4H, 8.46–8.50), (s, 8H, 9.09).

### 3.8. Synthesis of TCPP-Cysteine Disulfide (TCPP-Cysteine) (**7**)

To synthesize **7**, compound **6** (152 mg, 104 µmol) was combined with l-Cysteine hydrochloride (128 mg, 729 µmol) in DMF (5.2 mL). The solution was stirred at 60 °C for 48 h. The cysteine derivative was purified by precipitation in aqueous solution containing 25% EtOH followed by the addition HCl (180 µL, 37%/*v*). The reddish-brown material was washed several times with the same ethanolic solution and dried using a lyophilizer. The final product was stored at −20 °C. Yield: 124 mg, 80.0%. ^1^H-NMR: (300 MHz; *d*_6_-DMSO): δ_H_, ppm (m, 8H, 2.98–3.11), (m, 8H, 3.52–3.61), (m, 8H, 3.68–3.87), (m, 2H, 4.28–4.41), (m, 2H, 4.63–4.71), (m, 16H, 8.12–8.41), (s, 8H, 8.86).

### 3.9. Synthesis of Redox-Responsive TCPP Silane Derivative (RR-TCPP) (**8**)

To synthesize RR-TCPP, compound **7** (153 mg, 61 µmol) was combined with TES-PI (106 µL, 428 µmol) and Et_3_N (125 µL, 895 µmol) in anhydrous DMF (11 mL). The solution was stirred in an ice bath for 2 h under N_2_ conditions. The mixture was removed from the ice bath and stirred at room temperature for another 20 h still under N_2_ atmosphere. The redox-responsive ligand was purified by precipitation in H_2_O (60 mL) followed by the addition HCl (150 µL, 37%/*v*). The black powder was washed several times with the same aqueous solution and dried using a lyophilizer. The final product was stored at −20 °C. Yield: 152 mg, 65.0%. IR: 1019 cm^−1^ (Si–O), 1222 cm^−1^ (Si–C), 1714 cm^−1^ (carbamide).

### 3.10. Synthesis of TCPP-Ethyl Thiol (TCPP-EtSH) by Reduction of TCPP-PDSCA with dl-Dithiothreitol (DTT) (**9**)

To synthesize **9**, compound **6** (66.9 mg, 46 µmol) was combined with DTT (127 mg, 823 µmol) in DMF (6.5 mL). The solution was stirred at room temperature for 24 h. The thiol derivative was purified by precipitation in aqueous solution containing 25% EtOH followed by the addition HCl (60 µL, 37%/*v*). The brown solid was washed several times with the same solution and dried using a lyophilizer. The final product was stored at −20 °C. Yield: 31.5 mg, 67.0%. The successful synthesis of TCPP-EtSH was confirmed by the disappearance of the aromatic protons for the pyridine in the ^1^H-NMR. ^1^H-NMR: (300 MHz; *d*_6_-DMSO): δ_H_, ppm (q, 8H, 2.74–2.85), (m, 8H, 3.54–3.65), (m, 16H, 8.21–8.42), (s, 8H, 8.86).

### 3.11. Synthesis of 2 Pyridyl Disulfide Cysteamine (PDSCA) (**10**)

To synthesize PDSCA, cysteamine hydrochloride (1.132 g, 9.96 mmol) was dissolved in MeOH (10 mL) and added dropwise to a mixture of 2,2′-dipyridyl disulfide (4.4062 g, 20 mmol) and acetic acid (800 µL, 99%/*v*) in MeOH (20 mL) over 30 min. The mixture was stirred at room temperature for 24 h. The compound was purified by rotatory evaporation of MeOH followed by precipitation with diethyl ether. The white crystals were dried under high vacuum and stored at room temperature. Yield: 1.86 g, 84.0%. IR: 1608 cm^−1^ (aromatic); ^1^H-NMR: (300 MHz; *d*_6_-DMSO): δ_H_, ppm (m, 4H, 2.98–3.18), (t, 1H, 7.27–7.33), (d, 1H, 7.73–7.78), (t, 1H, 7.81–7.88), (s, 3H, 8.16–8.28), (d, 1H, 8.49–8.53); ^13^C-NMR: (300 MHz; *d*_6_-DMSO): δ_C_, ppm 35.30, 38.21 (aliphatic carbons), 120.60, 122.21, 138.49, 150. 40, 158.59 (aromatic carbons).

### 3.12. Singlet Oxygen (^1^O_2_) Determination for TCPP (**2**), TCPP-Serine (**4**) and TCPP-EtSH (**9**)

To measure the amount of ^1^O_2_ generated by **2**, **4**, and **9**, 40 μL of DPBF from a stock solution (8 mM, DMF) were dissolved in 4 mL of a DMF solution of photosensitizer (2.5 µM). The solution was irradiated with white light (400–700 nm, 41 mW/cm^2^) at different times (20, 40 and 60 s). The absorbance at 419 nm of these solutions was measured using a UV-vis spectrophotometer after illumination. Moreover, control experiments were run in the absence of light. In addition, experiments were carried out using red light (630–700 nm, 89 mW/cm^2^) following the same protocol. All the experiments were run by triplicate. The decrease from the original amount of DPBF was used to calculate the concentration of ^1^O_2_ produced.

### 3.13. Synthesis of C-TCPP- and RR-TCPP-PSilQ Nanoparticles

The synthesis of PSilQNPs was carried out through a reverse-microemulsion method. An organic phase was prepared mixing cyclohexane (7.5 mL), 1-hexanol (1.6 mL) and Trition X-100 (1.9 mL). At the same time, an aqueous solution containing C-TCPP (8 mg), NH_4_OH (4 mL) and H_2_O (4 mL) was prepared and immediately added to the organic phase solution dropwise. The mixture was allowed to stir at room temperature for 24 h. After that, the C-TCPP-PSilQNPs were obtained by crashing down the material after addition of EtOH (40 mL). The material was separated from the solution by centrifugation and washed twice with EtOH to get rid of any starting reagents. The final product was stored in EtOH. RR-TCPP-PSilQNPs were fabricated using the same protocol.

### 3.14. Singlet Oxygen (^1^O_2_) Determination for C-TCPP- and RR-TCPP-PSilQ Nanoparticles

To measure the amount of ^1^O_2_ generated by C-TCPP- and RR-TCPP-PSilQNPs, 40 μL of DPBF from a stock solution (8 mM, DMF) were dissolved in 4 mL of a DMF dispersion of PSilQNPs containing the equivalent amount of 2.5 µM of TCPP. The solution was irradiated with white light (400–700 nm, 41 mW/cm^2^) at different times (60 and 120 s). The absorbance at 419 nm of these solutions was measured using a UV-vis spectrophotometer after illumination. Moreover, control experiments were run in the absence of light. In addition, experiments were carried out using red light (630–700 nm, 89 mW/cm^2^) following the same protocol. All the experiments were run by triplicate. The decrease from the original amount of DPBF was used to calculate the concentration of ^1^O_2_ produced.

### 3.15. Photophysical Characterization of C-TCPP- and RR-TCPP-PSilQ Nanoparticles

A Cary 300 Bio UV/vis (Varian, Sidney, Australia) and a Cary Eclipse fluorescence spectrometers (Varian, Sidney, Australia) were used to determine the absorption and fluorescence emission of C-TCPP- and RR-TCPP-PSilQNPs, respectively. The nanoparticles were redispersed in DMF with a concentration of 0.5 mg/mL. TCPP (4 µM) dissolved in DMF was used as control sample.

### 3.16. Release Profile of TCPP-EtSH from RR-TCPP-PSilQNPs under High Reducing Environment

To determine the release of TCPP-EtSH compound under simulated reducing conditions, the reducing agent dithiothreitol (DTT) was used. The RR-TCPP-PSilQNPs were washed several times (at least five) with DMF to eliminate any physisorbed porphyrin. The nanoparticles were redispersed in 10 mL of DMF with a concentration of 0.35 mg/mL. Then, the dispersion was stirred for 9 h total under N_2_ atmosphere to determine the amount of background TCPP-EtSH. After that, DTT was dissolved in the dispersion to get a final concentration of DTT of 10 mM. Aliquots were taken at certain intervals of time and the absorption was measured to determine the amount of TCPP-EtSH molecules released. A similar procedure was followed for the control RR-TCPP-PSilQNPs that were only stirred in DMF (no addition of DTT).

### 3.17. In Vitro Phototoxicity of C-TCPP- and RR-TCPP-PSilQ Nanoparticles in Human Cervical Cancer (HeLa) Cells

HeLa cells were seeded at a density of 1 × 10^4^ cells/mL in a 96-well cell plates and incubated in 100 µL of RPMI-1640 cell media for 24 h at 37 °C. Cells were then inoculated with C-TCPP- and RR-TCPP-PSilQNPs (0.01, 0.05, 0.1, 0.5 and 1.0 µM of TCPP) for 24 h in cell media, followed by PBS washing steps, and then further incubated in PBS for light exposure. Samples were exposed to a LumaCare LC122 light source (630–700 nm; 89 mW/cm^2^) for 20 min. After irradiation, the cells were incubated in cell media for another 24 h and the cell survival was tested by the MTS assay (CellTiter 96^®^ AQueous Assay, Promega, Madison, WI, USA). The absorbance was measured at a wavelength of 450 nm in plate reader Multiskan FC. Cell viability percentage was calculated based on the absorbance measured relative to that of control culture cells.

## 4. Conclusions

We have developed a redox-responsive TCPP-PSilQNP platform for the transport and delivery of porphyrin-based photosensitizers with improved phototherapeutic effect toward human cervical cancer cells. TCPP-PSilQNPs are stable under simulated physiological conditions and exhibited a high content of PSs, 120–150 µmol of TCPP per g of PSilQNPs. The redox-responsive properties of the RR-TCPP-PSilQNPs were tested in solution using DTT as reducing agent. The phototoxic efficacy of these nanoparticles was evaluated *in vitro* using HeLa cells under light exposure by the MTS assay. RR-TCPP-PSilQNPs showed a higher phototoxicity than the control C-TCPP-PSilQNPs. Presumably, because of the efficient transport and intracellular release of TCPP-EtSH molecules. Moreover, TCPP-PSilQNPs contain carboxylic acid groups that can be further functionalized with polymers such as poly(ethylene glycol) and targeting agents to improve their targeting ability and therapeutic efficacy. TCPP-PSilQNP platform is a promising strategy in the fabrication of versatile photosensitizer nanocarriers with stimulus-responsive properties for oncological photodynamic therapy. Nevertheless, to move this PSilQNP system toward clinical applications, there are still several barriers that need to be overcome such as evaluating its efficacy, pharmacokinetics and biodistribution in animal models; its scalability and reproducibility following good manufacturing practices (GMP); and its biocompatibility and efficacy in clinical trials. Our group is currently testing the performance of this platform in animal models.

## Supplementary Materials

Supplementary materials can be found at http://www.mdpi.com/1422-0067/17/1/56/s1.
