# Supplementary material for: Redox-Responsive Porphyrin-Based Polysilsesquioxane Nanoparticles for Photodynamic Therapy of Cancer Cells
Source: Int J Mol Sci. 2015 Dec 31;17(1):56. doi: 10.3390/ijms17010056 (PMC4730301; doi:10.3390/ijms17010056)
Supplement: Supplementary file 1 [file ijms-17-00056-s001.pdf]

Supplementary Materials: Redox-Responsive Porphyrin-Based Polysilsesquioxane Nanoparticles for Photodynamic Therapy of Cancer Cells

Daniel L. Vega, Patrick Lodge and Juan L. Vivero-Escoto

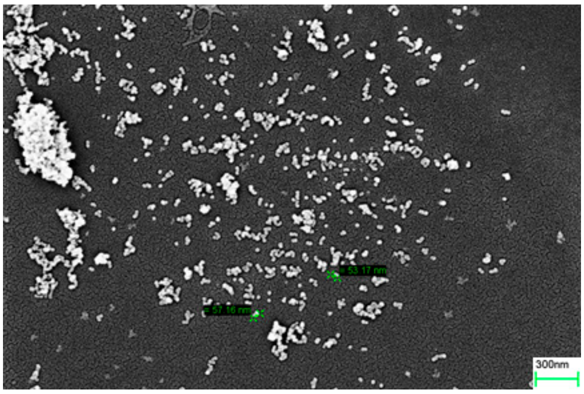

Figure S1. Scanning electron microscopy (SEM) image of redox-responsive tetrakis(carboxyphenyl) porphyrin polysilsesquioxane nanoparticles (RR-TCPP-PSilQNPs). Green marks indicate the size of individual nanoparticles. Scale bar = 300 nm.

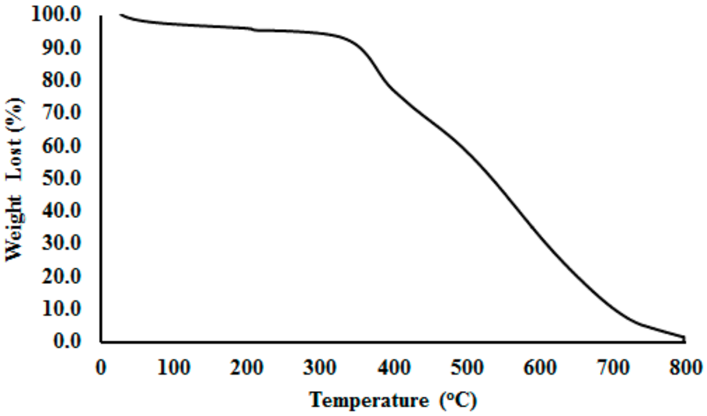

Figure S2. Thermogravimetric analysis of tetrakis(carboxyphenyl) porphyrin (TCPP) molecule.

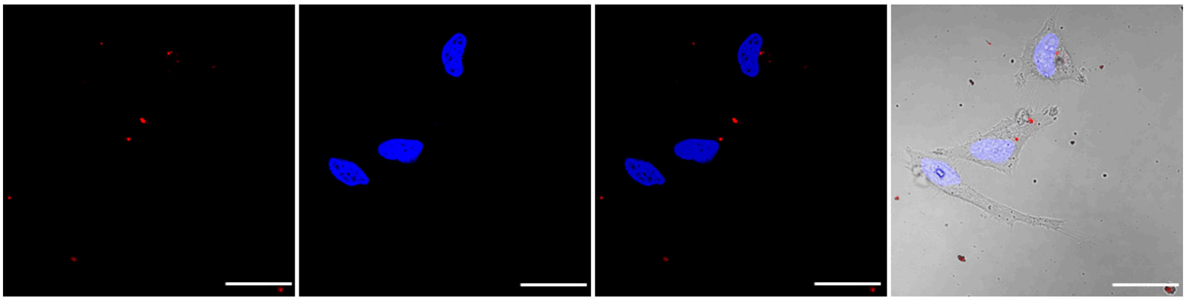

(**a**) (**b**) (**c**) (**d**)

Figure S3. Laser scanning confocal microscopy images of HeLa cells inoculated with RR-TCPP-PSilQNPs. Red fluorescence of the RR-TCPP-PSilQNPs (**a**); 4′,6-diamidino-2-phenylindole (DAPI)-stained nuclei (**b**); the overlapped imaged of red and DAPI-stained nuclei (**c**); and the overlapped imaged with the differential interference contrast (DIC) channel (**d**). Scale bars = 20 μm.

Experimental details for the confocal microscopy of RR-TCPP-PSilQNPs:

HeLa cells were seeded at a density of 5 × 10^4 cells/mL in a six-well culture plates with coverslips at the bottom of the wells and incubated in 3 mL of RPMI-1640 cell media for 24 h at 37 °C with 5% CO_2. The cell media was replaced by 3 mL of RR-TCPP-PSilQNPs (15 μg/mL) and incubated for 12 h in the RPMI-1640 cell media. Finally, the cell-plated coverslips were washed twice with PBS buffer (1 mM, pH 7.4) and stained with NucBlue® Live cell (ThermoFisher Scientific, Waltham, MA, USA) staining DAPI solution for 15 min. The stained coverslips were placed in microscope slides and examined under an Olympus Fluoview FV 1000 confocal fluorescence microscope system (Olympus America Inc., Center Valle, PA, USA).
